# Contrasting DCIS and invasive breast cancer by subtype suggests basal-like DCIS as distinct lesions

**DOI:** 10.1038/s41523-020-0167-x

**Published:** 2020-06-17

**Authors:** Helga Bergholtz, Tonje G. Lien, David M. Swanson, Arnoldo Frigessi, Oslo Breast Cancer Research Consortium (OSBREAC), Tone F. Bathen, Elin Borgen, Anne Lise Børresen-Dale, Olav Engebråten, Øystein Garred, Jürgen Geisler, Gry Aarum Geitvik, Olaf Johan Hartmann-Johnsen, Solveig Hofvind, Vessela N. Kristensen, Anita Langerød, Ole Christian Lingjærde, Gunhild Mari Mælandsmo, Bjørn Naume, Hege Russnes, Torill Sauer, Ellen Schlichting, Helle Kristine Skjerven, Maria Grazia Daidone, Jörg Tost, Fredrik Wärnberg, Therese Sørlie

**Affiliations:** 1 Department of Cancer Genetics, Institute for Cancer Research, Oslo University Hospital, Oslo, Norway; 2 Institute of Clinical Medicine, Faculty of Medicine, University of Oslo, Oslo, Norway; 3 Oslo Centre for Biostatistics and Epidemiology, Oslo University Hospital, Oslo, Norway; 4 Department of Biostatistics, University of Oslo, Oslo, Norway; 5 Department of Applied Research and Technical development, Fondazione IRCCS Istituto Nazionale dei Tumori, Milan, Italy; 6 Laboratory for Epigenetics and Environment, Centre National de Recherche en Génomique Humaine, CEA-Institut de Biologie Francois Jacob, Evry, France; 7 Department of Surgical Sciences, Uppsala University, Uppsala, Sweden; 8 Department of Surgery, Uppsala Academic Hospital, Uppsala, Sweden; 9 Department of Circulation and Medical Imaging, Norwegian University of Science and Technology (NTNU), Trondheim, Norway; 10 Department of Pathology, Division of Laboratory Medicine, Oslo University Hospital, Oslo, Norway; 11 Department of Oncology, Division of Cancer Medicine, Oslo University Hospital, Oslo, Norway; 12 Department of Tumor Biology, Institute for Cancer Research, Oslo University Hospital, Oslo, Norway; 13 Department of Oncology, Akershus University Hospital, Lørenskog, Norway; 14 Division of Medicine, Akershus University Hospital, Lørenskog, Norway; 15 Østfold Hospital, Østfold, Norway; 16 Cancer Registry of Norway, Oslo, Norway; 17 Oslo and Akershus University College of Applied Sciences, Faculty of Health Science, Oslo, Norway; 18 Department of Clinical Molecular Biology, Division of Medicine, Akershus University Hospital, Lørenskog, Norway; 19 Centre for Cancer Biomarkers CCBIO, Bergen, Norway; 20 Centre for Cancer Biomedicine, University of Oslo, Oslo, Norway; 21 Department of Computer Science, University of Oslo, Oslo, Norway; 22 Department of Pharmacy, Faculty of Health Sciences, University of Tromsø, Tromsø, Norway; 23 Department of Pathology, Akershus University Hospital, Lørenskog, Norway; 24 Section for Breast and Endocrine Surgery, Oslo University Hospital, Ullevål, Oslo, Norway; 25 Breast and Endocrine Surgery, Department of Breast and Endocrine Surgery, Vestre Viken Hospital Trust, Drammen, Norway

**Keywords:** Cancer genomics, Cancer genomics, Diagnostic markers

## Abstract

Ductal carcinoma in situ (DCIS) is a non-invasive type of breast cancer with highly variable potential of becoming invasive and affecting mortality. Currently, many patients with DCIS are overtreated due to the lack of specific biomarkers that distinguish low risk lesions from those with a higher risk of progression. In this study, we analyzed 57 pure DCIS and 313 invasive breast cancers (IBC) from different patients. Three levels of genomic data were obtained; gene expression, DNA methylation, and DNA copy number. We performed subtype stratified analyses and identified key differences between DCIS and IBC that suggest subtype specific progression. Prominent differences were found in tumors of the basal-like subtype: Basal-like DCIS were less proliferative and showed a higher degree of differentiation than basal-like IBC. Also, *core basal* tumors (characterized by high correlation to the basal-like centroid) were not identified amongst DCIS as opposed to IBC. At the copy number level, basal-like DCIS exhibited fewer copy number aberrations compared with basal-like IBC. An intriguing finding through analysis of the methylome was hypermethylation of multiple protocadherin genes in basal-like IBC compared with basal-like DCIS and normal tissue, possibly caused by long range epigenetic silencing. This points to silencing of cell adhesion-related genes specifically in IBC of the basal-like subtype. Our work confirms that subtype stratification is essential when studying progression from DCIS to IBC, and we provide evidence that basal-like DCIS show less aggressive characteristics and question the assumption that basal-like DCIS is a direct precursor of basal-like invasive breast cancer.

## Introduction

Ductal carcinoma in situ (DCIS) is a non-invasive, non-obligate precursor to invasive breast cancer (IBC) with low risk of progression^1^. As breast cancer screening has become widespread, more DCIS lesions are being detected^2–4^. Autopsy studies and studies on DCIS from non-treated patients show that many lesions, if left alone, will never progress to invasive disease^5–9^. However, there is currently no robust method to distinguish DCIS with invasive potential from those that may be left untreated. Furthermore, DCIS is a heterogeneous disease and may at time of diagnosis vary from indolent lesions to tumors on the verge of becoming invasive. Clinical, histopathological and molecular characteristics may also vary considerabely^10,11^. As a consequence of this uncertainty, treatment for DCIS is often extensive, resulting in substantial overtreatment^12–15^.

Knowledge on the underlying mechanisms of progression from DCIS to IBC is still limited. In order to select the optimal treatment strategy for a patient diagnosed with DCIS, it would be beneficial to determine the tumor’s invasive potential. Several studies have observed few genomic and epigenomic differences between DCIS and IBC^16–19^. However, most breast cancer progression studies have not taken into account the significance of molecular subtype in DCIS. For IBC, molecular subtypes have distinct characteristics and also provide valuable prognostic and predictive information^20^. In a previous study, we found evidence of subtype specific progression from DCIS to IBC suggesting that each molecular subtype undergoes a distinct evolutionary disease course^21^. In DCIS, grade and growth pattern provide some information on risk of recurrence, yet, there is still a need for more precise risk prediction^22–24^. For this purpose, the Oncotype DX Breast DCIS score has been developed to predict individual risk of recurrence after breast conserving surgery (BCS)^25^. This assay, however, does not take into account the vast heterogeneity of DCIS and the low risk group still experienced a relatively high risk of recurrence of 10% after 10 years^26^. Nevertheless, this score illustrates the potential of molecular-based assays for risk prediction in DCIS.

In this study, we explore the differences between DCIS and IBC in a subtype-specific manner using data from three genomic levels: Gene expression, DNA copy number and DNA methylation. We observed that DCIS and IBC of the luminal A subtype were overall highly similar, while for the basal-like subtype, DCIS might represent a different molecular entity than its invasive counterpart. We hypothesize that tumors of different molecular subtypes may have different modes of progression, and by comparing DCIS and IBC for each subtype separately, we gain insight into the mechanisms of breast cancer invasion and progression.

## Results

### Tumor characteristics and PAM50 subtyping

**Fig. 1:**
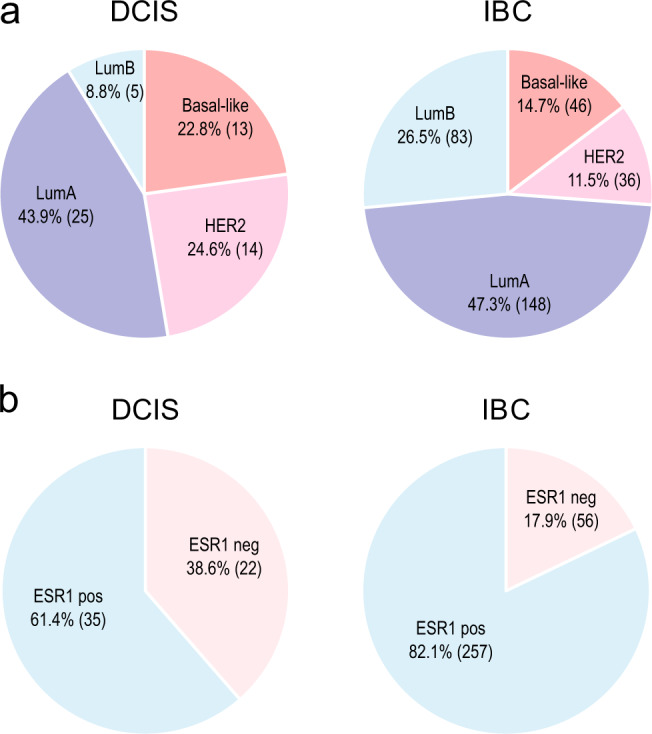
Distribution of PAM50 subtypes and ESR1 gene expression in DCIS and invasive breast cancer (IBC). **a** Distribution of PAM50 subtypes. **b** Distribution of ESR1 gene expression. Percentage of each subtype/ESR1-status is indicated, number of samples in parenthesis. There is significantly different distribution between DCIS and IBC for PAM50 subtypes (*P* = 0.0016 Fisher’s exact test) and ESR1 gene expression (*P* = 0.0012, Fisher’s exact test).

The study cohort includes data from 57 pure DCIS and 313 IBC cases. All samples were obtained from individual patients, i.e., none of the samples represents paired (synchronous) lesions from the same patient. DCIS lesions were from patients with no concurrent invasive disease (“pure” DCIS). All sample information including clinical and molecular parameters is presented in Table 1 and Supplementary Data 1. Based on expression of the PAM50 genes, we determined the intrinsic subtypes using the widely used centroid based classifier^27^ (see “Methods”), which provided correlation coefficients to each of the four centroids; basal-like, HER2-enriched, luminal A and luminal B. We found a significantly different distribution of the subtypes between DCIS and IBC (*P* = 0.0016, Fisher’s exact test, Fig. 1a). Most notably, there was a higher frequency of the HER2-enriched subtype and a lower frequency of Luminal B tumors in DCIS compared with IBC. This was reflected by a significantly different distribution of *ESR1* gene expression between DCIS and IBC (*P* = 0.0012 Fisher’s exact test, Fig. 1b). In general, we observed that DCIS tumors showed lower correlation coefficients to the subtype centroids compared with IBC; this was particularly evident for the basal-like subtype (Table 2). To investigate whether differences in tumor cell content could explain the lower subtype correlation coefficients in DCIS compared with IBC, we used ASCAT (Allele-Specific Copy number Analysis of Tumors)^28^ to calculate tumor purity based on copy number data (see Methods). We found no significant difference in tumor cell content between DCIS and IBC (Basal-like: *P* = 0.86, HER2: *P* = 0.13, LumA: *P* = 0.88, LumB: *P* = 0.19, Mann–Whitney *U* tests, Supplementary Fig. 1a).

**Table 1:** Summary of available data for analysis.

	DCIS	IBC
Number of tumors	57	313
Number of expression arrays	57	313
Number of SNP arrays	48	290
Number of Methylation arrays	41	273
Age in years, median (range)	54 (26–82)	54 (26–83)
Size in mm, median (range)	28 (7–90)	18 (2–130)
ELSTON grade (1/2/3/NA)	–	44/115/122/32
EORTC grade (1/2/3/NA)	0/8/21/28	−

ELSTON grading applies to invasive breast cancer (IBC). EORTC grading applies to DCIS (ductal carcinoma in situ).

**Table 2:** Median and range of subtype correlation coefficients to each PAM50 subtype for DCIS and invasive breast cancer (IBC).

	DCIS		IBC	
Subtype	Median	Range	Median	Range
Basal	0.26	(0.12–0.46)	0.76	(0.03–0.88)
HER2	0.35	(0.14–0.64)	0.55	(0.15–0.72)
LumA	0.50	(0.20–0.71)	0.56	(0.13–0.82)
LumB	0.33	(0.15–0.39)	0.45	(0.13–0.69)

### Diverging subtype characteristics between DCIS and IBC

The overall lower correlation to the PAM50 centroids in DCIS compared with IBC prompted us to explore the expression of the PAM50 genes in each subtype and tumor type to identify the contribution of each gene to the subtyping output (Supplementary Fig. 1b). Only one gene (Matrix metalloproteinase 11, *MMP11*, also named stromelysin 3) clearly delineated DCIS and IBC. *MMP11* is expressed in stromal cells and favors cancer cell survival and tumor progression through cleavage of collagen VI^29^. *MMP11* was markedly lower expressed in DCIS of all subtypes compared with IBC, in accordance with its non-invasive state. All other PAM50 genes showed expression patterns characteristic of the subtypes, independent of tumor type. Luminal genes (e.g., *ESR1, PGR, NAT1, BCL2, SLC39A6*) were higher expressed in luminal tumors in both DCIS and IBC compared with tumors of basal-like and HER2-enriched subtypes. Basal-like IBC showed markedly higher expression of genes associated with proliferation compared with all other subtypes (including basal-like DCIS). Both DCIS and IBC of the HER2-enriched subtype showed elevated expression of genes typically highly expressed in this subtype (*ERBB2, GRB7*, and *TMEM45B*). Of note, keratins associated with basal epithelium (*KRT5, KRT14*, and *KRT17*) were markedly higher expressed in DCIS of non-basal-like subtypes compared with their invasive counterpart while for the basal-like subtype, these keratins were highly expressed in both DCIS and IBC. This observation may be explained by gene expression contribution from a retained myoepithelial cell layer in DCIS.

**Fig. 2:**
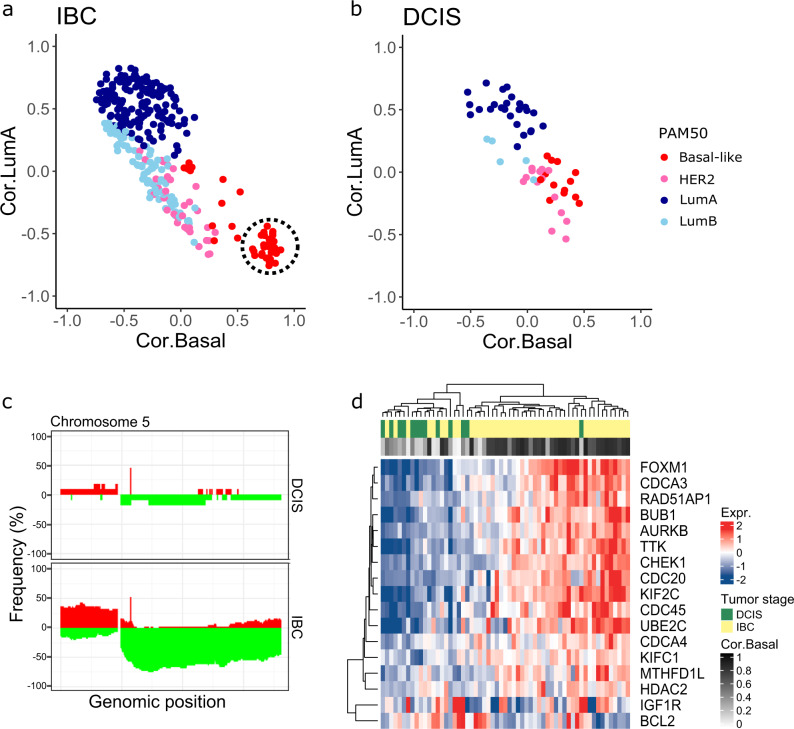
Core basal characteristics. Association between correlation coefficient to basal-like centroid on the *x*-axis and correlation coefficient to luminal A centroid on the *y*-axis for IBC (**a**) and DCIS (**b**). Core basal invasive tumors indicated by the dashed circle. **c** Frequency-plot of copy number data for IBC and DCIS. Genomic position is shown on the *x*-axis. The y-axis shows the frequency of losses (green) or amplifications (red) in DCIS and IBC, separately. **d** Heatmap showing expression of core basal genes in DCIS and IBC tumors of the basal-like subtype. The genes included have previously been shown to be correlated “in trans” with deletion of chr5q in core basal invasive breast cancer^31^.

Interestingly, we identified a distinct group of basal-like IBCs with high correlation to the basal-like centroid and correspondingly low correlation to the luminal A centroid (Fig. 2a), which was not found among basal-like DCIS (Fig. 2b). These invasive tumors may correspond to so-called *core basal* tumors, characterized by deletions on chromosome 5q and high expression of specific genes associated “in trans” with such deletions^30,31^. In accordance with this, we found 5q deletions at high frequency in basal-like IBC, while in only a minority of basal-like DCIS (Fig. 2c). Clustering gene expression values of the core basal-defining genes revealed two distinct clusters: one consisting of mostly IBC tumors with high correlation to the basal-like subtype (i.e. the core basal tumors), and a second cluster including most of the DCIS tumors and IBC tumors with low correlation to the basal-like subtype (Fig. 2d). By visual inspection of the distribution of the correlation coefficient to the basal-like centroid, we classified core basal tumors as those with correlation >0.6 (Fig. 2a, b). When investigating the PAM50 genes separately for the core and the non-core basal invasive tumors compared with basal-like DCIS, we found that the non-core basal invasive tumors showed lower expression of proliferation genes and higher expression of luminal genes compared with core basal invasive tumors (Supplementary Fig. 1c) Also, *EGFR* and basal keratins (which are known to be highly expressed in core basal tumors) showed lower expression in non-core basal tumors compared with core basal invasive tumors, while intermediate expressed in basal-like DCIS. The core basal invasive tumors were all estrogen receptor (ER) negative by immunohistochemistry (IHC) and by *ESR1* expression. Among the non-core basal invasive tumors, 8 out of 12 were *ESR1* expression positive. Five out of 13 basal-like DCIS were *ESR1* expression positive, however, there was no observable difference in correlation to the basal-like centroid between *ESR1* expression positive (*n* = 5) and *ESR1* expression negative (*n* = 8) basal-like DCIS (*P* = 0.62, Mann–Whitney *U* test).

### Extensive genomic differences between basal-like DCIS and basal-like IBC

We found few gene expression differences between DCIS and IBC when performing principal component analysis (PCA) of genome-wide gene expression data across all subtypes (Supplementary Fig. 2a). This is in accordance with previous studies^16,17^. However, after subtype stratification, PCA clearly separated IBC from DCIS in the basal-like and HER2-enriched subtypes, while not in the luminal subtypes (Supplementary Fig. 2b). Also, with respect to copy number aberrations, differences between DCIS and IBC varied between subtypes. DCIS exhibited overall fewer copy number changes compared with IBC as demonstrated by a lower genomic instability index (GII) in all subtypes, and the difference was significant for all subtypes except luminal B (Supplementary Fig. 3a and Supplementary Data 1). Nevertheless, the specific copy number changes in DCIS are reminiscent of invasive tumors, including 17q12 amplification in the HER2-enriched subtype and deletions of 16q in luminal A (Supplementary Fig. 4). Again, the largest difference between DCIS and IBC was found for basal-like tumors with DCIS showing substantially fewer copy number aberrations compared with basal-like IBC.

**Fig. 3:**
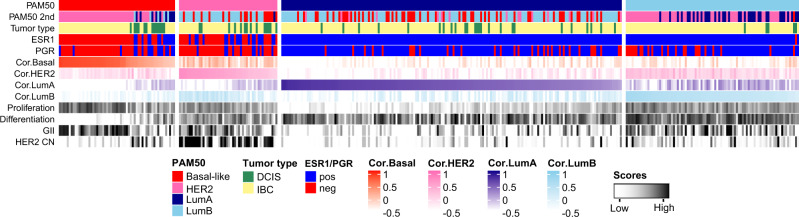
Genomic characteristics of DCIS and IBC. Each column represents one tumor. Columns are sorted according to PAM50 subtype and next, according to correlation to the tumor’s subtype. Relevant characteristics that commonly differ between molecular subtypes are shown and revealed pronounced differences between DCIS and IBC for the basal-like subtype. PAM50: The sample’s subtype. PAM50 2nd: The subtype with second highest correlation. Tumor type: DCIS (green). IBC (yellow). ESR1: Estrogen receptor 1 gene expression. PGR: Progesterone receptor gene expression. Cor.Basal/Cor.Her2/Cor.LumA/Cor.LumB: Correlation coefficients to the four PAM50 subtypes. Proliferation: Gene expression-based proliferation score. Differentiation: Gene expression-based differentiation score. GII: Genomic Instability Index based on copy number data. HER2 CN: HER2 copy number.

To further explore subtype specific differences between DCIS and IBC, we included information on the strength of the correlation to all other subtype centroids (Fig. 3, Supplementary Data 1). We found that basal-like IBC correlated highly to the basal-like centroid, and next, to the HER2-enriched centroid, while basal-like DCIS showed overall lower correlation to the basal-like centroid and more often had luminal subtypes as their second subtype (Fig. 3). On the contrary, luminal A tumors, both DCIS and IBC, showed relatively high correlation to the luminal A centroid and a similar distribution of the second best subtype (mostly basal-like and luminal B). Next, we calculated gene expression-based proliferation-, differentiation-, immune-, stromal-, and epithelial-to-mesenchymal transition (EMT)-scores, as well as HER2-copy number status (Fig. 3, Supplementary Fig. 3 and Supplementary Data 1). Both DCIS and IBC tumors showed subtype specific characteristics such as higher proliferation and lower differentiation in basal-like and HER2-enriched subtypes when compared with luminal A. In general, DCIS received lower stromal and EMT scores compared with IBC. The differences between DCIS and IBC were most pronounced in basal-like tumors: Basal-like DCIS displayed significantly lower median proliferation score compared with basal-like IBC (Supplementary Fig. 3b), while the median differentiation score was significantly higher in basal-like DCIS compared with IBC (Supplementary Fig. 3c), although still lower than in DCIS of any other subtype. Interestingly, there was no statistically significant difference in median immune score, median stromal score or median EMT score between basal-like DCIS and IBC (Supplementary Fig. 3d, e, f). The distinct difference seen between core and non-core basal invasive tumors prompted us to investigate these scores for core and non-core basal invasive tumors separately (Supplementary Fig. 5). For GII and proliferation, the scores for non-core basal invasive tumors were in between basal-like DCIS and core basal invasive tumors, while the differentiation scores were at the level of basal-like DCIS. There was no difference between core and non-core basal invasive tumors with regards to immune-, stromal- and EMT-scores. Overall, these findings show that subtype profiles of DCIS are comparable to those found in IBC, except for the basal-like subtype where DCIS appears to be associated with less aggressive gene expression characteristics.

### Long range epigenetic silencing of cPCDH genes occurs in basal-like IBC

**Fig. 4:**
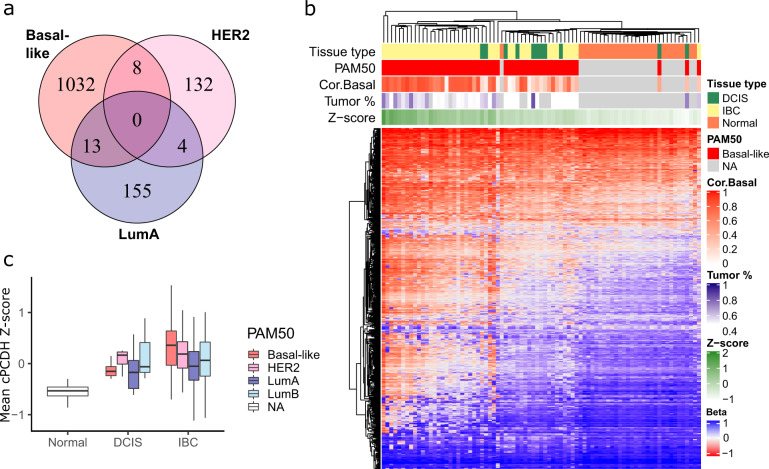
DNA methylation differences between DCIS and IBC. **a** Genes with significantly different methylation profiles between DCIS and IBC in basal-like, HER2-enriched and Luminal A subtypes (Mann–Whitney *U* test, FDR < 0.05 and effect size within the top 20%). No genes showed significantly differential methylation between DCIS and IBC for the luminal B subtype. **b** Heatmap showing methylation status (*β*-values) of all 698 CpGs in the 800 kb genomic window spanning the cPCDH genes on 5q in both tumor types (DCIS in green, IBC in yellow) and normal tissue samples in orange. PAM50 subtype, correlation to basal-like centroid (Cor.Basal), tumor percentage and mean cPCDH methylation (Z-score) are shown as column annotations. **c** Mean cPCDH methylation in normal and tumor tissue. The difference in mean cPCDH methylation between DCIS and IBC was significant for the basal-like subtype (*P* = 0.001, Mann–Whitney *U* test), while for the other subtypes, the difference between DCIS and IBC was not significant (HER2-enriched: *P* = 0.648, Luminal A: *P* = 0.233, Luminal B: *P* = 0.946, Mann Whitney *U* tests). Sample sizes: Basal-like (DCIS *n* = 11, IBC *n* = 41), HER2-enriched (DCIS *n* = 11, IBC *n* = 35), Luminal A (DCIS *n* = 16, IBC *n* = 125), Luminal B (DCIS *n* = 3, IBC *n* = 72). Boxplots illustrate the median (middle line) and the third and first quartiles (box); the whiskers indicate 1.5 × IQR above and below the box.

We identified numerous genes with significantly different methylation profiles between DCIS and IBC (Supplementary Data 2). For the basal-like subtype, 1053 genes showed statistically significant different methylation profile between DCIS and IBC, while for the HER2-enriched and luminal A subtypes, only 144 and 172 genes, respectively, showed significantly different methylation profiles (Fig. 4a). Due to low sample size, no genes with statistically significant different methylation profiles were identified for the luminal B subtype. None of the differentially methylated genes were common between the other three subtypes. Among the genes with significantly different methylation profiles between basal-like DCIS and IBC were multiple clustered protocadherins (cPCDH). These genes are involved in cell-cell adhesion and are organized in three clusters on chromosome 5q31 and notably; the genes are highly overlapping^32,33^. Long range epigenetic silencing (LRES) has previously been shown to occur in cancer in an 800 kb genomic window spanning the cPCDH gene clusters^34–36^. To corroborate the methylation profile analyses and explore whether LRES is characteristic of basal-like IBC, we clustered all basal-like tumors based on the β-values of the 698 CpGs present in this genomic window (Fig. 4b). For comparison, we also included normal breast tissue samples. This analysis revealed that basal-like invasive tumors with high correlation to the basal-like centroid were, in general, characterized by hypermethylation across the cPCDH genes, while normal samples displayed low levels of methylation. Basal-like DCIS showed significantly lower mean cPCDH methylation compared with basal-like IBC (*P* = 0.001, Mann–Whitney *U* test, Fig. 4c). Importantly, there was no association between mean cPCDH methylation and tumor percentage, indicating that the lower methylation levels of the cPCDHs in basal-like DCIS is not simply an artifact of normal tissue in these samples. The basal-like invasive tumors showed the highest cPCDH methylation levels of all tumors. Notably, the distinct difference between DCIS and IBC seen in the basal-like subtype was not found for any of the other subtypes (Fig. 4c). Of note, the highly overlapping organization of the cPCDH genes complicates interpretation of these results, since one CpG may be located in multiple genes simultaneously, e.g., in the transcription start site of one gene while in the gene body of other genes. This may in theory yield different effects on gene expression.

When compiling methylation, copy number and gene expression data of the cPCDHs for the basal-like tumors, it appeared that invasive tumors with hypermethylation of the cPCDH genes often exhibited deletions of the same genes, and that these changes corresponded well with correlation to the basal-like centroid (Supplementary Fig. 6). Importantly, the cluster of tumors with concurrent hypermethylation and deletion of the cPCDH genes consisted mainly of aneuploid tumors, while the sub-cluster containing most DCIS consisted only of diploid tumors. We could not detect any effect of hypermethylation or 5q deletions on cPCDH gene expression. This could possibly be explained by expression of retained alleles in polyploid tumors or by post-transcriptional regulation. In summary, the notable differences in cPCDH methylation between basal-like DCIS and IBC support our previous results that basal-like DCIS may be a different entity than basal-like IBC.

## Discussion

In this study, we have explored differences between DCIS and IBC in a subtype specific manner using gene expression, copy number and DNA-methylation data derived from fresh frozen tumor material. The study was instigated by findings from our previous study where we hypothesized that progression of DCIS to invasive cancer differ between molecular subtypes^21^. The indolent nature of many in situ tumors and the fact that many of these tumors never progress to invasive or metastatic disease correlate poorly with the results from several studies showing remarkably few genomic differences between DCIS and IBC^16–18^. This lack of genomic dissimilarity may be explained by inherent differences between the molecular subtypes: In most breast cancer cohorts, the majority of tumors are of luminal subtypes; hence, characteristics that differentiate between DCIS and IBC in unstratified analyses are confounded by subtypes. The different distribution of molecular subtypes observed between IBC and DCIS may in part be explained by underrepresentation of small DCIS lesions and, consequently, overrepresentation of high-grade DCIS lesions included in the cohort. However, the frequency of tumors of the least aggressive subtype (luminal A) is similar in DCIS and IBC, indicating that the observed difference in subtype distribution between the two tumor types represents a true distinction.

Interestingly, the most pronounced differences between DCIS and IBC were found for the basal-like subtype. Basal-like DCIS showed lower correlation to the basal-like centroid (i.e., low “basalness”) compared with basal-like IBC, and there were no core basal DCIS in our data. This is in accordance with a previous integrative clustering analysis that showed genomic isolation of basal-like IBC, and not basal-like DCIS^37^. In the present study we showed that the basal-like DCIS tumors exhibited higher correlation to Luminal A subtype, higher degree of differentiation, lower proliferation and lower genomic instability than basal-like IBC. Also with respect to alterations of DNA methylation, basal-like tumors did prominently show more differences between DCIS and IBC compared with all other subtypes. Most notable was the marked hypermethylation of CpGs mapping to the (cPCDHs) genes in basal-like IBC compared with DCIS and a positive association between hypermethylation of cPCDHs and degree of “basalness”. Hypermethylation of DNA in the genomic location spanning the cPCDH genes through long range epigenetic silencing (LRES)^38^ has been shown to increase with progression of cervical cancer^36^ and has also been observed in breast cancer^34^, colorectal cancer^35^ and Wilm’s tumor^39^. Interestingly, the chromosomal region of the cPCDH genes (5q31) is frequently deleted in basal-like IBCs and is a defining feature of core basal IBC tumors^40,41^. cPCDHs are molecules involved in cell-cell adhesion and have also been shown to inhibit cell growth and suppress oncogenic pathways, features consistent with a role as tumor suppressors^42^. Loss of intraepithelial cell-cell adhesion is a key feature during tumor cell invasion^43,44^ and it is tempting to speculate that loss of cPCDH tumor suppressor function through LRES may contribute to driving the invasion process specifically in basal-like cancer.

During tumor evolution, transition from DCIS to an invasive stage may represent an evolutionary bottleneck which may also impact tumor subtype^1,45^. To study subtype evolution and plasticity during tumor progression and invasion, we would need consecutive biopsies from the same patients. Nonetheless, our study includes sufficient number of samples to be able to compare subtype characteristics between DCIS and IBC as groups for each subtype, separately. We show that the difference between DCIS and IBC is greater for the basal-like subtype compared with all other subtypes. Despite that the intrinsic subtypes were defined in IBC, we believe that basal-like DCIS are truly basal-like since firstly, the PAM50 subtyping showed that they correlate the most to the basal-like centroid, albeit to a lower degree than IBC. Secondly, several genomic features of basal-like tumors are also present in basal-like DCIS, including low degree of differentiation, high expression of basal keratins, low expression of luminal genes and expression of genes indicative of immune cell infiltration. Despite these similarities, basal-like DCIS may not be precursors to basal-like IBC. Basal-like breast cancer is an aggressive disease that develops rapidly. Especially the core basal tumors have an aggressive phenotype with poorer prognosis than non-core basal tumors^30,46^. Although all core basal invasive tumors at some point must have progressed from an intraductal stage, the transition from DCIS to IBC may occur so rapidly that the probability of “capturing” such tumors as DCIS is very small, as also proposed by Kurbel^47^. This hypothesis is supported by the fact that basal-like invasive breast tumors have fewer concurrent DCIS lesions compared with other subtypes^48,49^. Our results indicate that DCIS in general possesses characteristics that resemble those of invasive tumors of the same subtype. It is therefore uncontroversial to hypothesize that a DCIS with basal-like characteristics will progress to a basal-like cancer with its well-known characteristics. However, our results indicate that many basal-like DCIS resemble the less aggressive non-core basal invasive tumors and hence, we therefore speculate that patients diagnosed with basal-like DCIS do not carry high-risk tumors. Potentially may they be slow-growing tumors that never progress to an invasive tumor in the life-time of the patient^50^. This may have profound impact on how we perceive DCIS and not least, how they should be treated.

A limitation of this study is the lack of follow-up information on recurrence or survival. Hence, our results need to be validated in a DCIS cohort with more extensive clinical follow-up information. Also, the subtype stratified approach that we have employed, reduces the number of samples in each group which may preclude statistically significant results. The limited availability of small and low-grade DCIS for molecular analysis may artificially skew the cohort towards large or high-grade DCIS that may not be representative of the DCIS present in the population. Nevertheless, our study has reaffirmed the necessity of taking a subtype specific approach when studying progression of DCIS and we have demonstrated that there are substantial differences between basal-like DCIS and IBC that may question basal-like DCIS as precursor lesions to invasive breast carcinoma.

## Methods

### Tissue samples

This study includes gene expression, DNA copy number and DNA methylation data from 57 DCIS and 313 IBC cases. All samples were obtained from individual patients, i.e. none of the samples represents paired (synchronous) lesions from the same patient. DCIS lesions are from patients with no concurrent invasive disease (“pure” DCIS). Samples were fresh frozen tissue collected from three different patient cohorts, of which two (“Uppsala” and “Oslo2”) are previously published^51–56^. The third cohort, (“Milan”) has not been previously published and includes fresh frozen tissue from a total of 34 breast tumors. Histopathological evaluation of H&E stained tissue sections was performed by a trained pathologist. Normal breast tissue samples were obtained as core biopsies from women without breast cancer^57^. All women provided a signed informed consent for future biomarker research studies. This study complies with the Declaration of Helsinki, and was approved by the each institution’s internal review and ethics board (approval numbers: 2016/433 (Oslo, Norway), PG/U-25/01/2012-00001497 (Milan, Italy), 2005/118 (Uppsala, Sweden).

### DNA and RNA isolation

Total RNA and DNA was isolated using the QIAcube system with the AllPrep DNA/RNA Universal Kit (cat.no. 80224, Qiagen, Hilden, Germany) with 30 mg tissue as input. The tissue was manually minced with a scalpel on ice followed by homogenization using TissueLyzer LT and Qiashredder (Qiagen). RNA and DNA extraction was performed according to the protocol provided by the supplier. Nucleic acid concentrations were measured on a NanoDrop ND-1000 spectrophotometer (Thermo Scientific, Wilmington, DE, USA) and RNA integrity was analyzed using Agilent 2100 Bioanalyzer (Agilent Technologies, Santa Clara, USA).

### Gene expression analysis

To obtain whole genome expression data^58^, Agilent Sureprint G3 Human Gene Expression 8 × 60 K microarrays (G4851A) (Agilent, Technologies, Santa Clare, USA) with the Low Input Quick Amp Labeling protocol were used. RNA input was 40 ng and Cy3 was used as fluorophore. Quality Control (QC) was performed in Agilent’s Feature Extraction software. From the Milan cohort, five invasive breast carcinomas and 28 DCIS were successfully analyzed and passed all quality control criteria while one DCIS failed QC. As a control, one sample of commercially available normal breast RNA (Ambion Human Breast Total RNA, Thermo Fisher Scientific, Wilmington, DE, USA) was included throughout the whole experimental pipeline. The same microarray platform had been used for the two other patient cohorts. Data from all three cohorts were normalized together using quantile normalization. For genes represented with more than one probe, mean expression was calculated to obtain one gene expression value per gene.

### Genome-wide methylation

DNA methylation data^59^ was obtained using the Illumina Infinium HumanMethylation450K microarray (Illumina, Inc. CA, USA) following the manufacturer’s instructions. Data was preprocessed using subset quantile normalization^60^. The resulting *β* value represents the fraction of methylated DNA molecules at a specific CpG. Quality control of *β* values was performed as presented by Wilhelm-Benartzi et al.^61^: *β*-values with detection p-values higher than 0.05 (0.225% of the *β*-values) were replaced by NA. CpG sites where more than 25% of the *β* values failed quality control, were removed from the analysis resulting in 436 162 reliable CpGs in the final dataset. NA values were imputed using the R-function impute.knn with default parameters.

For the initial part of the analysis we obtained methylation profiles by performing PCA separately for each gene. All CpGs within the gene or 50 kB upstream or downstream of the gene were included. The value of the first principal component represents the gene’s methylation profile. This method allows for obtaining one value per gene per sample, while preserving as much information as possible from the CpGs representing each gene.

### Copy number aberrations analysis

Copy number data^62^ was obtained using Affymetrix SNP 6.0 arrays (Affymetrix, Santa Clara, CA, USA) at Aros Applied Biotechnology (Aarhus, Denmark) following the manufacturer’s instructions. CEL-files were processed using the PennCNV-Affy library^63^ with the HapMap samples as reference set^64^ and corrected for GC content^65^. The data was segmented using the PCF algorithm with arguments *k*_min_ = 5, gamma = 100 in the R copynumber package^66^. The copy number of the segment overlapping the gene the most was set as a gene’s copy number. Ploidy and tumor percentage were calculated using ASCAT^28^. In short, ASCAT can accurately dissect the allele-specific copy number of solid tumors, and simultaneously estimate both tumor ploidy and non-aberrant cell admixture. Genome instability index (GII) was derived by calculating the fraction of the genome affected by copy number change.

### PAM50 centroid-based subtype method for breast cancer

PAM50 subtyping, as described in Parker et al.^27^, uses gene centered expression data from 50 genes. Using Spearman correlation, we correlated gene expression data for each tumor sample to the published centroids and assigned the subtype with the highest correlation coefficient. Note that this PAM50 classifier requires the cohort to have a similar proportion of ER-positive tumors as the original training cohort^67^. In the training cohort, about 60% of tumors are ER-positive and gene centering for each gene can be described as follows:

$${\mathrm{Mean}}_{{\mathrm{all}}\;{\mathrm{patients}}} = 0.6 \bullet {\mathrm{Mean}}_{\mathrm{ER} + \mathrm{patients}} + 0.4 \bullet {\mathrm{Mean}}_{\mathrm{ER} - \mathrm{patients}}$$

Since the composition of ER-positive patients is higher than 60% in cohorts included in this study, we adjusted our cohort to the training cohort, by calculating the mean for the ER-positive and ER-negative tumors separately, before calculating the overall mean according to the formula above. ER-status was determined by using the *ESR1* gene expression value which showed a distinct bimodal distribution enabling a reliable cut-off to be set. Consistency in ER status derived by IHC and *ESR1* expression was high, with 98% of the tumors (320/327) concurring. Progesterone receptor (PR) status was derived by *PGR*-expression the same way as for ER (Supplementary Data 1).

### Gene expression-based tumor scores

Proliferation scores were calculated using an 11-gene proliferation signature^68^ and EMT scores were calculated using an EMT signature based on four adhesion genes (weighted negatively) and seven EMT-genes (weighted positively) (Supplementary Data 1): For each gene and sample, a standard (Z) score was calculated, then the proliferation/EMT-scores were obtained for every tumor by calculating the mean of all Z-scores across all genes in the signature. Differentiation scores were derived using the differentiation predictor described in Prat et al.^69^ and immune and stromal infiltration scores were calculated using ESTIMATE^70^.

### Differential methylation

Genes differentially methylated between DCIS and IBC where identified using Mann–Whitney *U* tests separately for each subtype. False discovery rate was used to correct for multiple testing. Cut-offs for identifying differentially methylated genes were set at both FDR and effect size (defined as the absolute difference in median between DCIS and IBC) to increase the likelihood of finding the biological relevant differences between the two groups. We included genes with FDR < 0.05 and effect size within the top 20% (corresponds to a cut-off > 0.127). Mean cPCDH methylation was calculated for each tissue sample (tumor and normal tissue) as the mean of standard (Z) scores for all relevant CpGs.

### Statistical and bioinformatic analyses

All statistical analyses were conducted in R^71^ unless otherwise specified. Heatmaps were created using the R package Complex Heatmaps^72^ and other plots were created using the package ggplot2^73^. Fisher exact tests were used to compare distribution of subtype and ER-status between the two tumor types. Mann–Whitney *U*-tests (two-sided) were used to compare tumor content, GII, proliferation scores, differentiation scores, immune scores, stromal scores, EMT scores and mean cPCDH methylation between DCIS and IBC separately for each subtype. Correlation between cPCDH methylation and tumor percentage was calculated using spearman correlation.

### Reporting summary

Further information on experimental design is available in the Nature Research Reporting Summary linked to this paper.

## Supplementary information

Supplementary figures

Supplementary Data 1

Supplementary Data 2

Reporting summary

## Data Availability

The data generated and analyzed during this study are described in the following metadata record: 10.6084/m9.figshare.12293102^74^. Gene expression, copy number and DNA methylation data from Oslo2 and Uppsala tumor cohorts and DNA methylation data from normal tissue samples, analyzed during this study, have previously been published and are publicly available at Gene Expression Omnibus: https://identifiers.org/geo:GSE80999^53^, https://identifiers.org/geo:GSE59248^55^, https://identifiers.org/geo:GSE60185^54^, and at the European Genome-phenome Archive (EGA): https://identifiers.org/ega.dataset:EGAD00010000942^56^. The data of the Milan cohort, generated during this study, are available at the European Genome-phenome Archive (EGA): https://identifiers.org/ega.dataset:EGAD00010001863^62^ (DNA copy number data), https://identifiers.org/ega.dataset:EGAD00010001864^58^ (gene expression data) and https://identifiers.org/ega.dataset:EGAD00010001865^59^ (DNA methylation data). Due to the European general data protection regulations, the processed datasets in.Rdata file format are not publicly available, but can be made available on reasonable request from the corresponding author, Dr. Therese Sørlie, email: tsorlie@rr-research.no. To access the data, researchers must complete an institutional agreement, and it must be verified that the research to be conducted is covered by the current study’s ethical approval and the patients’ consents.

## Code availability

Details on the code used in this study are available upon request.

## References

[1] Cowell CF (2013). Progression from ductal carcinoma in situ to invasive breast cancer: revisited. Mol. Oncol..

[2] Seely JM, Alhassan T (2018). Screening for breast cancer in 2018—what should we be doing today?. Curr. Oncol..

[3] Virnig BA, Tuttle TM, Shamliyan T, Kane RL (2010). Ductal carcinoma in situ of the breast: a systematic review of incidence, treatment, and outcomes. J. Natl Cancer Inst..

[4] Ernster VL (2002). Detection of ductal carcinoma in situ in women undergoing screening mammography. J. Natl Cancer Inst..

[5] Sanders ME, Schuyler PA, Dupont WD, Page DL (2005). The natural history of low-grade ductal carcinoma in situ of the breast in women treated by biopsy only revealed over 30 years of long-term follow-up. Cancer.

[6] Page DL, Dupont WD, Rogers LW, Landenberger M (1982). Intraductal carcinoma of the breast: follow-up after biopsy only. Cancer.

[7] Page DL, Dupont WD, Rogers LW, Jensen RA, Schuyler PA (1995). Continued local recurrence of carcinoma 15–25 years after a diagnosis of low grade ductal carcinoma in situ of the breast treated only by biopsy. Cancer.

[8] Nielsen M, Jensen J, Andersen J (1984). Precancerous and cancerous breast lesions during lifetime and at autopsy. A study of 83 women. Cancer.

[9] Collins LC (2005). Outcome of patients with ductal carcinoma in situ untreated after diagnostic biopsy: results from the Nurses’ Health Study. Cancer.

[10] Burstein HJ, Polyak K, Wong JS, Lester SC, Kaelin CM (2004). Ductal carcinoma in situ of the breast. N. Engl. J. Med..

[11] Gorringe KL, Fox SB (2017). Ductal carcinoma in situ biology, biomarkers, and diagnosis. Front. Oncol..

[12] Esserman LJ (2014). Addressing overdiagnosis and overtreatment in cancer: a prescription for change. Lancet Oncol..

[13] Narod SA, Iqbal J, Giannakeas V, Sopik V, Sun P (2015). Breast cancer mortality after a diagnosis of ductal carcinoma in situ. JAMA Oncol..

[14] Groen EJ (2017). Finding the balance between over- and under-treatment of ductal carcinoma in situ (DCIS). Breast.

[15] Sagara Y, Julia W, Golshan M, Toi M (2017). Paradigm shift toward reducing overtreatment of ductal carcinoma In situ of breast. Front. Oncol..

[16] Ma X-J (2003). Gene expression profiles of human breast cancer progression. Proc. Natl Acad. Sci. USA.

[17] Vincent-Salomon A (2008). Integrated genomic and transcriptomic analysis of ductal carcinoma in situ of the breast. Clin. Cancer Res..

[18] Hwang ES (2004). Patterns of chromosomal alterations in breast ductal carcinoma in situ. Clin. Cancer Res..

[19] Fleischer T (2014). Genome-wide DNA methylation profiles in progression to in situ and invasive carcinoma of the breast with impact on gene transcription and prognosis. Genome Biol..

[20] Sørlie T (2001). Gene expression patterns of breast carcinomas distinguish tumor subclasses with clinical implications. Proc. Natl Acad. Sci. USA.

[21] Lesurf R (2016). Molecular features of subtype-specific progression from ductal carcinoma in situ to invasive breast cancer. Cell Rep..

[22] Wang SY, Shamliyan T, Virnig BA, Kane R (2011). Tumor characteristics as predictors of local recurrence after treatment of ductal carcinoma in situ: a meta-analysis. Breast Cancer Res. Treat..

[23] Wallis MG (2012). The effect of DCIS grade on rate, type and time to recurrence after 15 years of follow-up of screen-detected DCIS. Br. J. Cancer.

[24] Onega T (2017). The diagnostic challenge of low-grade ductal carcinoma in situ. Eur. J. Cancer.

[25] Rakovitch E (2015). A population-based validation study of the DCIS Score predicting recurrence risk in individuals treated by breast-conserving surgery alone. Breast Cancer Res. Treat..

[26] Hanna WM (2019). Ductal carcinoma in situ of the breast: an update for the pathologist in the era of individualized risk assessment and tailored therapies. Mod. Pathol..

[27] Parker J (2009). Supervised risk predictor of breast cancer based on intrinsic subtypes. J. Clin. Oncol..

[28] Van Loo P (2010). Allele-specific copy number analysis of tumors. Proc. Natl Acad. Sci. USA.

[29] Motrescu ER (2008). Matrix metalloproteinase-11/stromelysin-3 exhibits collagenolytic function against collagen VI under normal and malignant conditions. Oncogene.

[30] Tischkowitz M (2007). Use of immunohistochemical markers can refine prognosis in triple negative breast cancer. BMC Cancer.

[31] Curtis C (2012). The genomic and transcriptomic architecture of 2,000 breast tumours reveals novel subgroups. Nature.

[32] Gul IS, Hulpiau P, Saeys Y, van Roy F (2017). Evolution and diversity of cadherins and catenins. Exp. Cell Res..

[33] Chen WV, Maniatis T (2013). Clustered protocadherins. Development.

[34] Novak P (2008). Agglomerative epigenetic aberrations are a common event in human breast cancer. Cancer Res..

[35] Dallosso AR (2012). Long-range epigenetic silencing of chromosome 5q31 protocadherins is involved in early and late stages of colorectal tumorigenesis through modulation of oncogenic pathways. Oncogene.

[36] Wang KH (2015). Global methylation silencing of clustered proto-cadherin genes in cervical cancer: Serving as diagnostic markers comparable to HPV. Cancer Med.

[37] Swanson, D. M., Lien, T., Bergholtz, H., Sørlie, T. & Frigessi, A. A Bayesian two-way latent structure model for genomic data integration reveals few pan-genomic cluster subtypes in a breast cancer cohort. *Bioinformatics*10.1093/bioinformatics/btz381 (2019). 10.1093/bioinformatics/btz381 31077301

[38] Forn M (2013). Long range epigenetic silencing is a trans-species mechanism that results in cancer specific deregulation by overriding the chromatin domains of normal cells. Mol. Oncol..

[39] Dallosso AR (2009). Frequent long-range epigenetic silencing of protocadherin gene clusters on chromosome 5q31 in Wilms’ tumor. PLoS Genet..

[40] Bergamaschi A (2006). Distinct patterns of DNA copy number alteration are associated with different clinicopathological features and gene-expression subtypes of breast cancer. Genes Chromosom. Cancer.

[41] Yu W, Kanaan Y, Baed YK, Gabrielson E (2009). Chromosomal changes in aggressive breast cancers with basal-like features. Cancer Genet. Cytogenet..

[42] Van Roy F (2014). Beyond E-cadherin: roles of other cadherin superfamily members in cancer. Nat. Rev. Cancer.

[43] Huang RYJ, Guilford P, Thiery JP (2012). Early events in cell adhesion and polarity during epithelialmesenchymal transition. J. Cell Sci..

[44] Gheldof, A. & Berx, G. Cadherins and epithelial-to-mesenchymal transition. *Prog. Mol. Biol. Transl. Sci.* **116**, 317–336 (2013). 10.1016/B978-0-12-394311-8.00014-5 23481201

[45] Visser LL (2019). Discordant Marker expression between invasive breast carcinoma and corresponding synchronous and preceding DCIS. Am. J. Surg. Pathol..

[46] Cheang MCU (2008). Basal-like breast cancer defined by five biomarkers has superior prognostic value than triple-negative phenotype. Clin. Cancer Res..

[47] Kurbel S (2013). In search of triple-negative DCIS: tumor-type dependent model of breast cancer progression from DCIS to the invasive cancer. Tumor Biol..

[48] Doebar SC (2016). Extent of ductal carcinoma in situ according to breast cancer subtypes: a population-based cohort study. Breast Cancer Res. Treat..

[49] Badve S (2011). Basal-like and triple-negative breast cancers: a critical review with an emphasis on the implications for pathologists and oncologists. Mod. Pathol..

[50] Welch HG, Black WC (2010). Overdiagnosis in cancer. J. Natl Cancer Inst..

[51] Muggerud AA (2010). Molecular diversity in ductal carcinoma in situ (DCIS) and early invasive breast cancer. Mol. Oncol..

[52] Aure MR (2017). Integrative clustering reveals a novel split in the luminal A subtype of breast cancer with impact on outcome. Breast Cancer Res..

[53] Aure, M. R. et al. Integrative clustering reveals a novel split in the luminal A subtype of breast cancer with impact on outcome. *Gene Expression Omnibus* https://identifiers.org/geo:GSE80999 (2017). 10.1186/s13058-017-0812-y PMC5372339 28356166

[54] Fleischer, T. et al. Genome-wide DNA methylation profiles in progression to in situ and invasive carcinoma of the breast with impact on gene transcription and prognosis. *Gene Expression Omnibus* https://identifiers.org/geo:GSE60185 (2014). 10.1186/s13059-014-0435-x PMC4165906 25146004

[55] Lesurf, R. et al. Molecular features of subtype-specific progression from ductal carcinoma in situ to invasive breast cancer. *Gene Expression Omnibus* https://identifiers.org/geo:GSE59248 (2016). 10.1016/j.celrep.2016.06.051 27396337

[56] Sørlie, T. et al. Breast lesions assayed with Affymetrix SNP 6.0. *European Genome-phenome Archive* https://identifiers.org/ega.dataset:EGAD00010000942 (2016).

[57] Haakensen VD (2010). Expression levels of uridine 5’-diphospho-glucuronosyltransferase genes in breast tissue from healthy women are associated with mammographic density. Breast Cancer Res.

[58] Sørlie, T. et al. Gene expression. Milan samples. *European Genome-phenome Archive* https://identifiers.org/ega.dataset:EGAD00010001864 (2020).

[59] Sørlie, T. et al. DNA Methylation. Milan samples. *European Genome-phenome Archive* https://identifiers.org/ega.dataset:EGAD00010001865 (2020).

[60] Touleimat N, Tost J (2012). Complete pipeline for Infinium® Human Methylation 450K BeadChip data processing using subset quantile normalization for accurate DNA methylation estimation. Epigenomics.

[61] Wilhelm-Benartzi CS (2013). Review of processing and analysis methods for DNA methylation array data. Br. J. Cancer.

[62] Sørlie, T. et al. DNA Copy Number. Milan samples. *European Genome-phenome Archive* https://identifiers.org/ega.dataset:EGAD00010001863 (2020).

[63] Wang K (2007). PennCNV: an integrated hidden Markov model designed for high-resolution copy number variation detection in whole-genome SNP genotyping data. Genome Res..

[64] The International HapMap Consortium. (2003). The international HapMap project. Nature.

[65] Diskin SJ (2008). Adjustment of genomic waves in signal intensities from whole-genome SNP genotyping platforms. Nucleic Acids Res..

[66] Nilsen G (2012). Copynumber: Efficient algorithms for single- and multi-track copy number segmentation. BMC Genomics.

[67] Zhao X, Rødland EA, Tibshirani R, Plevritis S (2015). Molecular subtyping for clinically defined breast cancer subgroups. Breast Cancer Res..

[68] Nielsen TO (2010). A comparison of PAM50 intrinsic subtyping with immunohistochemistry and clinical prognostic factors in tamoxifen-treated estrogen receptor-positive breast cancer. Clin. Cancer Res..

[69] Prat A (2010). Phenotypic and molecular characterization of the claudin-low intrinsic subtype of breast cancer. Breast Cancer Res.

[70] Yoshihara K (2013). Inferring tumour purity and stromal and immune cell admixture from expression data. Nat. Commun..

[71] R Core Team & R Foundation for Statistical Computing. R: a language and environment for statistical computing. (2017).

[72] Gu Z, Eils R, Schlesner M (2016). Complex heatmaps reveal patterns and correlations in multidimensional genomic data. Bioinformatics.

[73] Wickham, H. *Ggplot2: elegrant graphics for data analysis*. (2016).

[74] Bergholtz, H. et al. Metadata supporting the published article: Contrasting DCIS and invasive breast cancer by subtype suggests basal-like DCIS as distinct lesions. *figshare*10.6084/m9.figshare.12293102 (2020). 10.1038/s41523-020-0167-x PMC7299965 32577501

