# Supplementary material for: Contrasting DCIS and invasive breast cancer by subtype suggests basal-like DCIS as distinct lesions
Source: NPJ Breast Cancer. 2020 Jun 17;6:26. doi: 10.1038/s41523-020-0167-x (PMC7299965; doi:10.1038/s41523-020-0167-x)
Supplement: Supplementary file 1 — Supplementary figures [file 41523_2020_167_MOESM1_ESM.pdf]

*Supplementary figures*

# Contrasting DCIS and invasive breast cancer by subtype suggests basal-like DCIS as distinct lesions

Helga Bergholtz, Tonje G. Lien, David M. Swanson, Arnoldo Frigessi, Oslo Breast Cancer Research Consortium (OSBREAC), Maria Grazia Daidone , Jörg Tost, Fredrik Wärnberg, Therese Sørlie

# Supplementary Figure 1

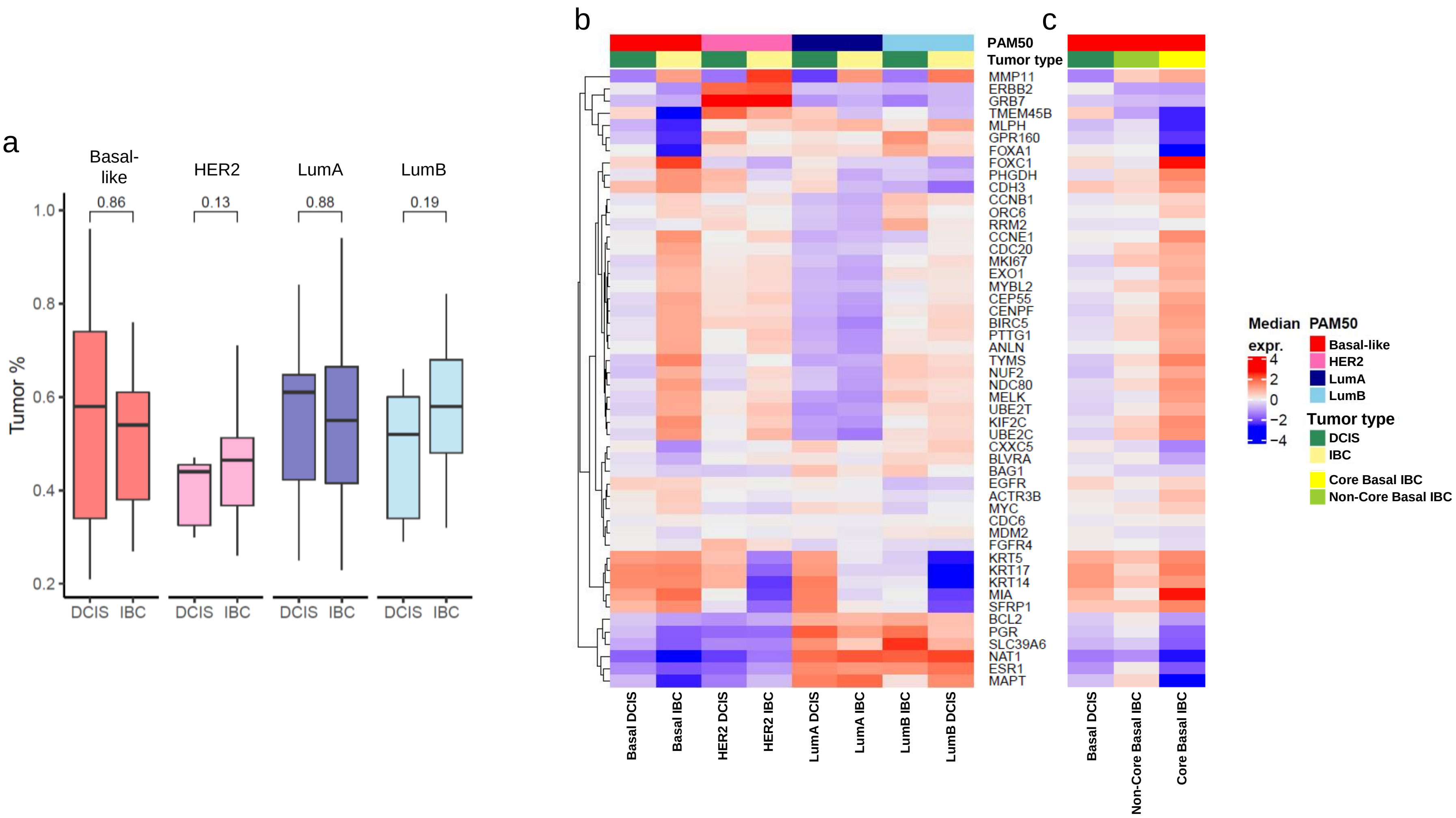

**Supplementary Figure 1. Tumor content and PAM50 gene expression in DCIS and IBC stratified by subtype.**
**(a)** Boxplots of tumor cell content derived from copy number data separated by tumor type and PAM50 subtype. P-values obtained by Mann Whitney U tests, DCIS vs. IBC in each subtype separately. Boxplots illustrate the median (middle line) and the third and first quartiles (box); the whiskers indicate 1.5×IQR above and below the box. (**b and c)** Heatmaps showing median gene expression values for genes included in the PAM50 centroid for each subtype and tumor type separately (b) and for basal-like DCIS, and core and non-core basal invasive tumors (c). Sample sizes: Basal-like (DCIS n=11, IBC n=44), HER2-enriched (DCIS n=12, IBC n=34), Luminal A (DCIS n=20, IBC n=136), Luminal B (DCIS n=5, IBC n=76).

# Supplementary Figure 2

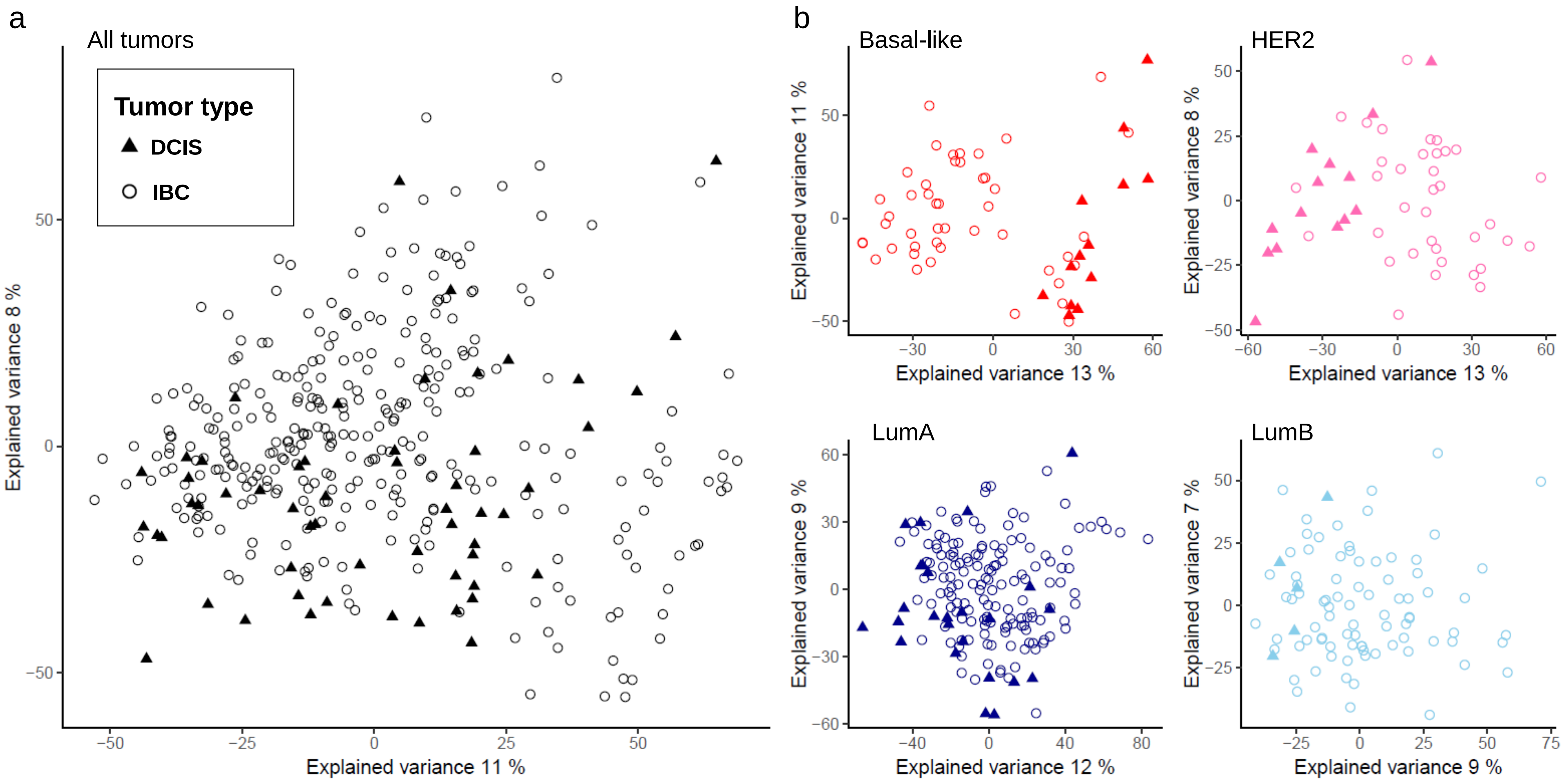

**Supplementary Figure 2. Principal Component Analysis based on genome-wide gene expression.**
Data of all samples **(a)** and separately for each of the four PAM50 subtypes **(b)**. The first principal component is shown on the x-axis and the second principal component on the y-axis.

# Supplementary Figure 3

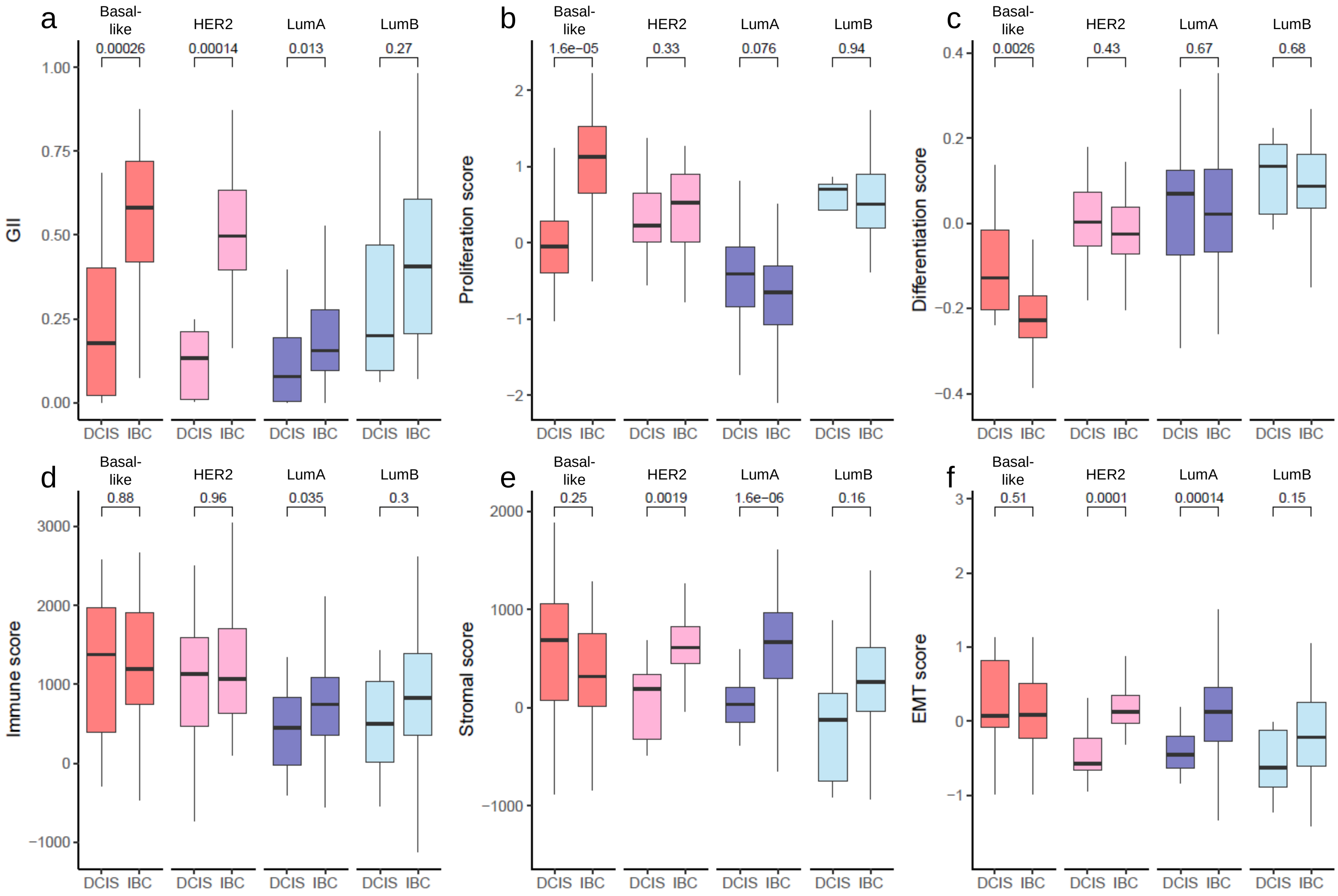

**Supplementary Figure 3. Genomic characteristics of DCIS and IBC of different subtypes.**
Boxplots showing genomic instability score (GII) based on copy number **(a)**, gene expression-based proliferation score **(b)**, differentiation score **(c)**, immune score **(d)**, stromal score **(e)** and EMT score **(f)** for DCIS and IBC, separated by subtype. P-values were obtained by Mann Whitney U tests, DCIS vs. IBC in each subtype separately. Sample sizes: Basal-like (DCIS n=13, IBC n=46), HER2-enriched (DCIS n=14, IBC n=36), Luminal A (DCIS n=25, IBC n=148), Luminal B (DCIS n=5, IBC n=83). Boxplots illustrate the median (middle line) and the third and first quartiles (box); the whiskers indicate 1.5×IQR above and below the box.

# Supplementary Figure 4

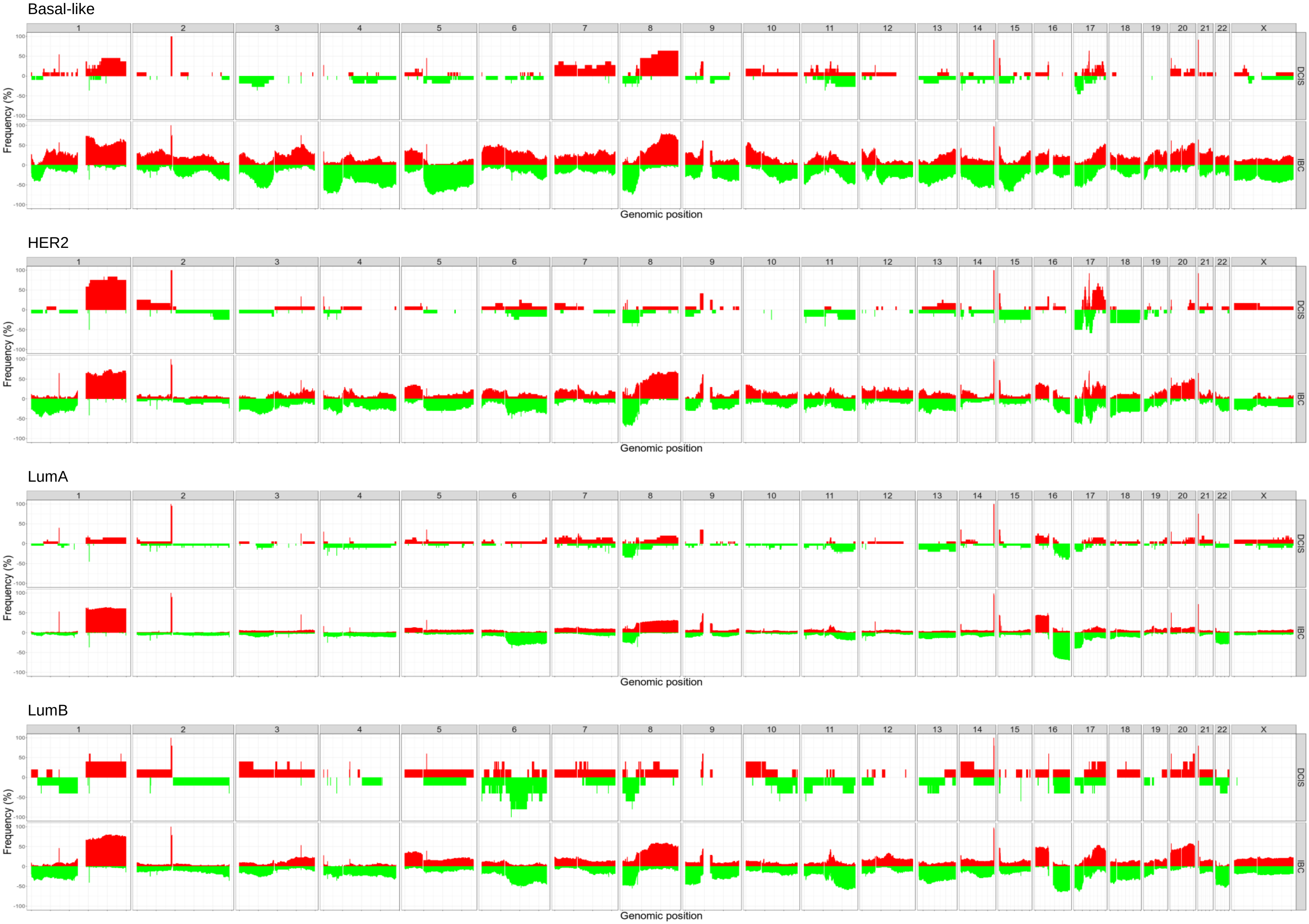

**Supplementary Figure 4. Frequency-plot of copy number aberrations in DCIS and IBC separately for the PAM50 subtypes.**
The x-axis shows the genomic position and the y-axis shows the frequency of losses (green) or amplifications (red).

# Supplementary Figure 5

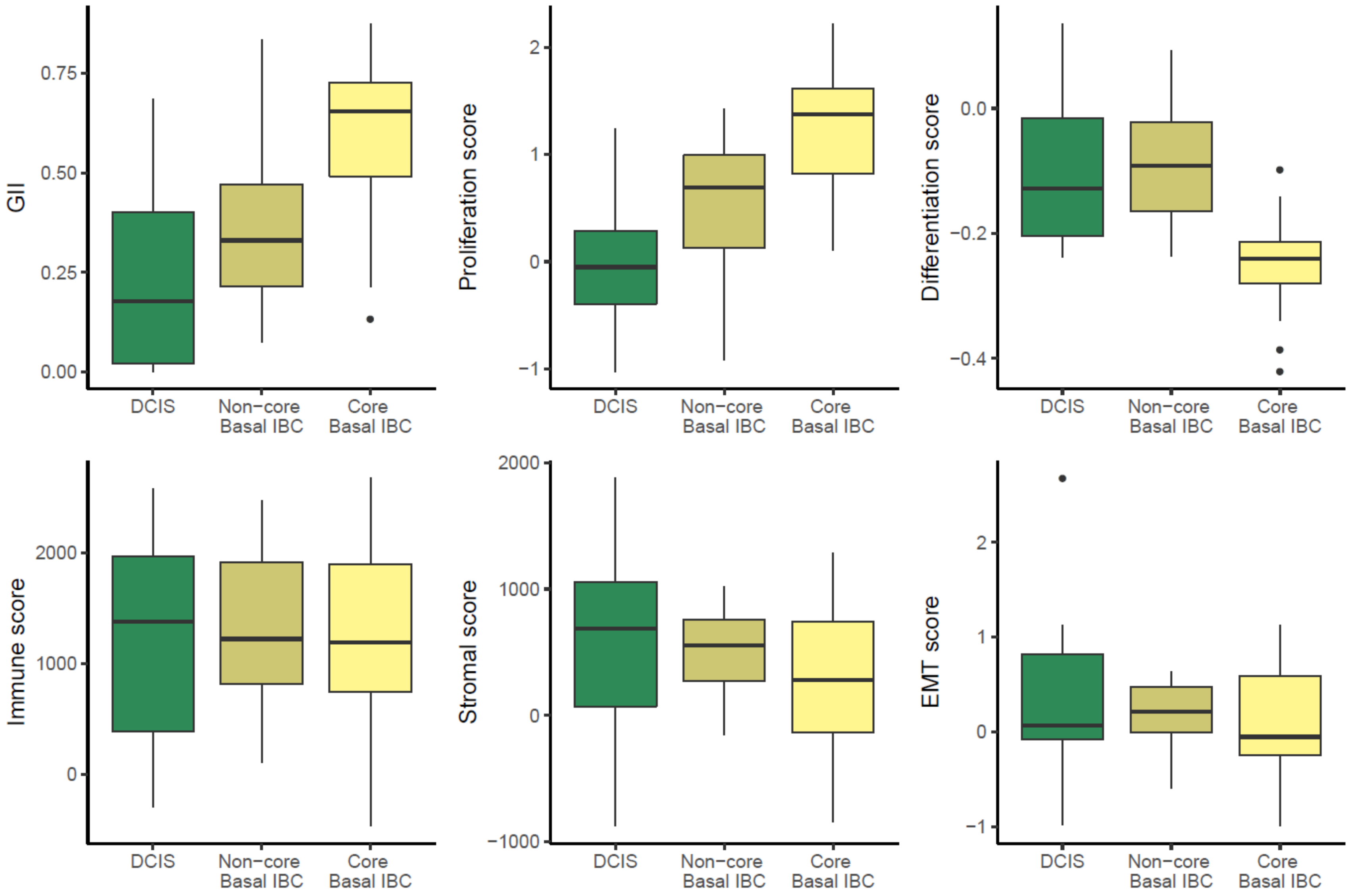

**Supplementary Figure 5. Genomic characteristics of basal-like DCIS and core/non-core basal-like IBC.**
Boxplots showing genomic instability score (GII) based on copy number (a), gene expression-based proliferation score (b), differentiation score (c), immune score (d), stromal score (e) and EMT score (f) for basal-like DCIS, and core and non-core basal invasive tumors. Sample sizes: Basal-like DCIS n=13, core basal IBC n=34, non-core basal invasive n=12. Boxplots illustrate the median (middle line) and the third and first quartiles (box); the whiskers indicate 1.5×IQR above and below the box; dots indicate outliers outside the range of the whiskers.

# Supplementary Figure 6

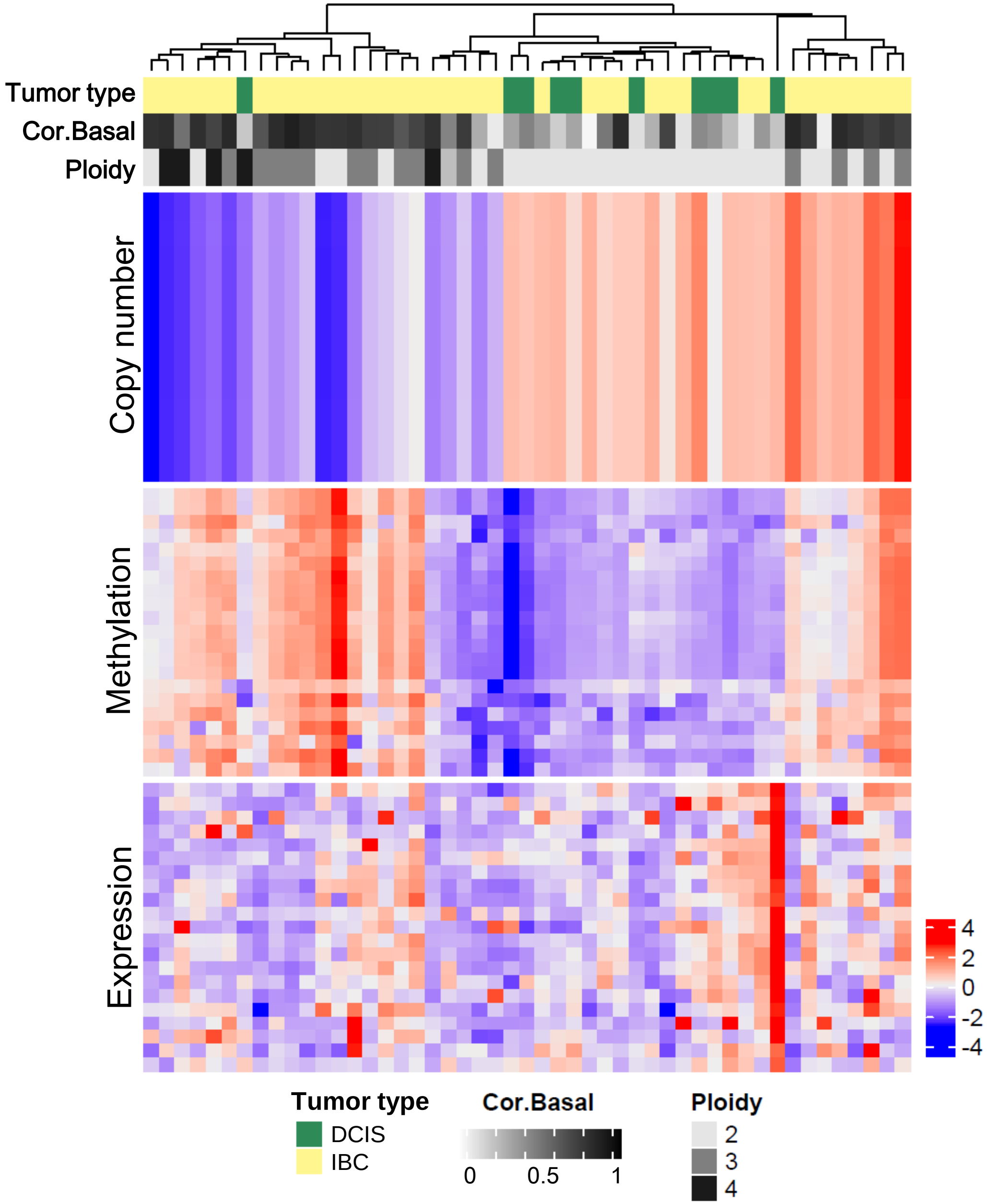

**Supplementary Figure 6. cPCDH copy number, DNA methylation and gene expression.**
The 21 cPCDHs that were significant differentially methylated between basal-like DCIS and basal-like IBC are shown across three genomic levels (copy number, methylation and gene expression). The genes are plotted in the same order in all three panels and samples are clustered across all genomic levels.

# Supplementary data captions

**Supplementary Data 1**
**Worksheet 1:** Sample annotations and calculated signature scores
**Worksheet 2:** List of genes included in proliferation and EMT scores

**Supplementary Data 2**
**Lists of genes with differentially methylated profiles between DCIS and IBC in Basal-like, HER2-enriched and Luminal A subtypes.**
Genes with FDR<0.05 (Mann-Whitney U test) and effect size within top 20% (corresponds to a cut-off of >0.127) are included.
